# Measurable residual disease monitoring by ddPCR in the early posttransplant period complements the traditional MFC method to predict relapse after HSCT in AML/MDS: a multicenter retrospective study

**DOI:** 10.1186/s12967-024-05114-w

**Published:** 2024-04-30

**Authors:** Weihao Chen, Jingtao Huang, Yeqian Zhao, Luo Huang, Zhiyang Yuan, Miner Gu, Xiaojun Xu, Jimin Shi, Yi Luo, Jian Yu, Xiaoyu Lai, Lizhen Liu, Huarui Fu, Chenhui Bao, Xin Huang, Zhongzheng Zheng, He Huang, Xiaoxia Hu, Yanmin Zhao

**Affiliations:** 1 Bone Marrow Transplantation Center of The First Affiliated Hospital & Liangzhu Laboratory, Zhejiang University School of Medicine, No.79 Qingchun Road, Hangzhou, China; 2 Institute of Hematology, Zhejiang University, Hangzhou, China; 3 Zhejiang Province Engineering Research Center for Stem Cell and Immunity Therapy, Hangzhou, China; 4 State Key Laboratory of Medical Genomics, Shanghai Institute of Hematology, National Research Center for Translational Medicine, Shanghai Rui Jin Hospital, Shanghai Jiao Tong University School of Medicine, No.197 Ruijiner Road, Shanghai, 200025 China; 5 Collaborative Innovation Center of Hematology, Shanghai Jiao Tong University School of Medicine, Shanghai, 200025 China; 6 Shanghai Dishuo Beken Biotechnology Co., Ltd, Shanghai, China; 7 Division of Hematology-Oncology, Children’s Hospital Zhejiang University School of Medicine, Hangzhou, Zhejiang China; 8 Zhejiang Province Key Laboratory of Hematology Oncology Diagnosis and Treatment, Hangzhou, China

**Keywords:** MRD, Allo-HSCT, ddPCR, MFC, Relapse

## Abstract

**Background:**

Droplet digital PCR (ddPCR) is widely applied to monitor measurable residual disease (MRD). However, there are limited studies on the feasibility of ddPCR-MRD monitoring after allogeneic hematopoietic stem cell transplantation (allo-HSCT), especially targeting multiple molecular markers simultaneously.

**Methods:**

Our study collected samples from patients with acute myeloid leukemia (AML) or high-risk myelodysplastic syndrome (MDS) in complete remission after allo-HSCT between January 2018 and August 2021 to evaluate whether posttransplant ddPCR-MRD monitoring can identify patients at high risk of relapse.

**Results:**

Of 152 patients, 58 (38.2%) were MRD positive by ddPCR within 4 months posttransplant, with a median variant allele frequency of 0.198%. The detectable *DTA* mutations (*DNMT3A*, *TET2*, and *ASXL1* mutations) after allo-HSCT were not associated with an increased risk of relapse. After excluding *DTA* mutations, patients with ddPCR-MRD positivity had a significantly higher cumulative incidence of relapse (CIR, 38.7% vs. 9.7%, *P* < 0.001) and lower rates of relapse-free survival (RFS, 55.5% vs. 83.7%, *P* < 0.001) and overall survival (OS, 60.5% vs. 90.5%, *P* < 0.001). In multivariate analysis, ddPCR-MRD positivity of non-*DTA* genes was an independent adverse predictor for CIR (hazard ratio [HR], 4.02; *P* < 0.001), RFS (HR, 2.92; *P* = 0.002) and OS (HR, 3.12; *P* = 0.007). Moreover, the combination of ddPCR with multiparameter flow cytometry (MFC) can further accurately identify patients at high risk of relapse (F+/M+, HR, 22.44; *P* < 0.001, F+/M-, HR, 12.46; *P* < 0.001 and F-/M+, HR, 4.51; *P* = 0.003).

**Conclusion:**

ddPCR-MRD is a feasible approach to predict relapse after allo-HSCT in AML/MDS patients with non-*DTA* genes and is more accurate when combined with MFC.

**Trial registration:**

ClinicalTrials.gov identifier: NCT06000306. Registered 17 August 2023 –Retrospectively registered (https://clinicaltrials.gov/study/NCT06000306?term=NCT06000306&rank=1).

**Supplementary Information:**

The online version contains supplementary material available at 10.1186/s12967-024-05114-w.

## Introduction

Allogeneic hematopoietic stem cell transplantation (allo-HSCT) is considered the only cure for acute myeloid leukemia (AML) or high-risk myelodysplastic syndrome (MDS), but the high mortality rate from relapse after transplantation remains a concern. Data from the CIBMTR show that over 30% of deaths following allo-HSCT in patients are caused by relapse [1]. The monitoring of measurable residual disease (MRD) after induction and consolidation therapy or intensive therapy (such as allo-HSCT) in patients with AML/MDS is a valuable predictor of relapse and survival [2, 3]. MRD detection can routinely identify subclinical levels of leukemia cells before clinical relapse and guide preventive or preemptive intervention to improve long-term survival [4, 5]. Currently, a variety of methods have been applied to detect MRD, including the determination of leukemia-associated immunophenotype (LAIP) using multiparameter flow cytometry (MFC), real-time qPCR with or without reverse transcription (RT-qPCR), next-generation sequencing (NGS) and droplet digital PCR (ddPCR) [4–7]. However, no uniform approach to detect MRD has yet been established, especially after allo-HSCT.

An optimal tool for posttransplant MRD monitoring requires high sensitivity, great repeatability, and the ability to accurately predict prognosis. MFC technology is widely used for MRD detection to predict patient outcomes [8–12], but its accuracy (detection depth to 0.01%) needs to be improved. In addition, this approach necessitates analytical and technical expertise. ddPCR, as a third-generation PCR, is a promising technology for absolute quantification of nucleic acids developed in recent years, with ultrahigh sensitivity (detection depth down to 0.001%) [13–15]. Therefore, the application of ddPCR to monitor MRD is an attractive choice to track disease remission, provide prognostic information, and guide clinical decision-making. The markers of MRD detection generally depend on the specific gene mutations and/or fusion gene transcripts identified through NGS at the time of diagnosis.

Published reports have shown that ddPCR-MRD monitoring is effective in predicting relapse when using specific single genes as MRD markers, such as *BCR-ABL*, *NPM1*, *PML-RARA*, *IDH1/2*, or *WT1* [16–20]. However, few studies have reported on the applicability of ddPCR in MRD monitoring after allo-HSCT, even targeting multiple molecular markers simultaneously [21]. Moreover, it is commonly accepted that age-related clonal hematopoiesis genes, especially *DNMT3A*, *TET2*, and *ASXL1* (*DTA*), have limited ability to predict prognosis when *DTA* is detectable before HSCT [22–25]. When *DTA* mutations are monitored as MRD markers during the posttransplant period, their prognostic impact remains controversial [26, 27]. This study aimed to evaluate whether posttransplant MRD monitoring by ddPCR can accurately distinguish patients with AML/MDS at high risk of relapse and to determine the prognostic role of *DTA* mutations after allo-HSCT in AML/MDS. Additionally, we performed simultaneous MRD analysis using ddPCR and MFC for the first time to determine whether combined detection can improve prediction accuracy.

## Materials and methods

### Study cohorts

**Fig. 1:**
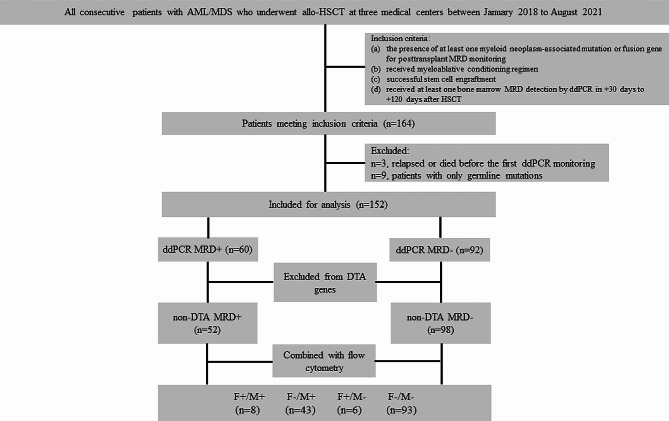
Consort diagram

The study reviewed 646 patients with AML/MDS who underwent allo-HSCT in three medical centers (the First Affiliated Hospital of Zhejiang University School of Medicine, Children’s Hospital of Zhejiang University School of Medicine and Shanghai Rui Jin Hospital) from January 2018 to August 2021. The inclusion criteria for this study were as follows: (a) the presence of at least one myeloid neoplasm-associated mutation or fusion gene detected at diagnosis by NGS or real-time qPCR provided for posttransplant MRD monitoring; (b) received myeloablative conditioning regimen; (c) successful stem cell engraftment; and (d) received at least one bone marrow MRD detection by ddPCR in + 30 days to + 120 days after HSCT. Patients who relapsed or died before the first ddPCR monitoring or patients with only germline mutations were excluded. A total of 152 patients who met the criteria were included in this study, comprising those with persistent MRD positivity of core binding factor (CBF) fusion genes and high-risk MDS before transplantation (ClinicalTrials.gov identifier: NCT06000306, a multicenter retrospective cohort study). The grouping process of the whole study is shown in Fig. 1. All patients signed written informed consent for this study. The entire study procedure was conducted according to the Declaration of Helsinki and was approved by the Ethics Review Committee of each center.

### MRD detection by ddPCR

The ddPCR primers and probes used for MRD detection mentioned in this article were designed and tested by Shanghai Dishuo Beken Biotechnology Co., Ltd. The primers, probe design, and detection were consistent with previously published studies of the company [28, 29]. DNA/RNA samples were extracted from the patients’ marrow aspirate using a QIAamp DNA/RNA blood Mini Kit according to the manufacturer’s instructions. All tumor-associated mutations or fusion genes were detected by a QuantStudio**™** 3D Digital PCR System (ABI), and VIC- and FAM-labelled probes were used to label mutant/fusion genes and wild type, respectively. QuantStudio**™** 3D AnalysisSuite**™** Relative Quantification Software (ABI) was used for data analysis. A template DNA sample without target mutations or fusion genes was used as a negative control, and each sample was repeated three times. The Poisson distribution was used to calculate the absolute copy numbers of mutation or non-mutation of each gene. ddPCR-MRD was quantified as the ratio of mutant numbers to the total numbers of wild type and mutant type. More than 1 genetically abnormal cell detected in 10,0000 cells (variant allele frequency, VAF ≥ 0.001%) by the ddPCR method can be considered as MRD positivity.

### MRD detection by MFC

MFC was performed on bone marrow samples to detect MRD at every follow-up after allo-HSCT. MRD detection by MFC used an 8- to 10-color assay according to previous publications [30–32]. MRD positivity was considered when a cluster of > 20 cells was identified that expressed two or more LAIP markers at diagnosis. For those patients who did not have LAIP markers, MRD was identified as a cell population that was different from the normal pattern of antigen expression in a specific cell lineage at a specific maturation stage compared with normal or regenerated bone marrow [33, 34]. The sensitivity of the MRD assay was 0.01%. A minimum of 200 000 events were routinely collected for MRD analysis. An isotype control monoclonal antibody was used. Samples were acquired on a three laser Navios instrument (Beckman Coulter, Fullerton, CA, USA). Data were analysed with Kaluza software (Beckman Coulter).

### Statistical analyses

The cumulative incidence was estimated for relapse (CIR), being competing risks with non-relapse mortality (NRM). The competing risk model using Gray test was applied as there are a piece of competing risks. OS and relapse-free survival (RFS) curves were plotted using the Kaplan‒Meier method. The log-rank test was applied for univariate comparisons of groups in OS and RFS.

Continuous variables were compared using the Mann‒Whitney U test or t test, while the comparisons of categorical variables were determined by the chi-square or Fisher’s exact test. All variables with *P* < 0.1 in univariate analysis were further included in multivariate analysis. In multivariate analysis, Cox proportional hazard regression model was adopted for OS and RFS, while the Fine-Gray proportional hazard regression model was constructed for CIR and NRM because of the competing risks.

The positive predictive value (PPV) was calculated by the ratio of true-positive patients / (true-positive + false-positive patients), and the negative predictive value (NPV) was calculated by the ratio of true-negative patients / (true-negative + false-negative patients) [35]. PPV and NPV indicate how many of the samples predicted as positive/negative by the tool are true positives/negative. These two indicators were employed to assess the precision of the binary categorical tool.

*P* < 0.05 was considered statistically significant. All statistical analyses were performed using SPSS version 26 (Chicago, IL, USA) and R software (version 4.2.1, R Foundation for Statistical Computing, Vienna, Austria).

## Results

### Patient characteristics and clinical outcome

A total of 152 patients, all of whom had at least one molecular marker and underwent ddPCR-MRD detection within 120 days after allo-HSCT, were included in our study cohort. In these patients, 525 ddPCR-MRD analyses were performed after allo-HSCT, with a median of 4 ddPCR-MRD analyses per patient (range 1 to 6 times). The median age of these patients was 44 years (range 12 to 67 years). The primary diseases included acute myeloid leukemia (AML; 83.6%, 127/152) and myelodysplastic syndrome (MDS; 16.4%, 25/152). There was no significant difference in baseline characteristics between the *DTA* MRD-positive group and *DTA* MRD-negative group. However, among patients with non-*DTA* genes, a higher risk level of the refined disease risk index (DRI-R) [36] was frequently observed in the MRD positivity group. The detailed patient characteristics are summarized in Table 1. Ultimately, 29 patients (19.1%) experienced relapse after allo-HSCT, with a median time to relapse of 8.1 months (range 2.5 to 20.6 months). Eight patients (5.3%) died from non-relapse disease.

**Table 1:** Comparison of clinical characteristics between MRD + and MRD- patients according to *DTA* genes and non-*DTA* genes

**Characteristic**	**All patients** **(N = 152)**	***DTA*** genes			Non-***DTA*** genes		
		MRD positive (n = 13)	MRD negative (n = 27)	***P*** Value	MRD positive (n = 52)	MRD negative (n = 98)	***P*** Value
**Median age (range)**	44(12–67)	50(21–64)	48(23–61)	0.194	43(13–64)	43(12–67)	0.442
**Sex, n (%)**				0.577			0.742
Male	78(51.3)	7(53.8)	12(44.4)		28(53.8)	50(51.0)	
Female	74(48.7)	6(46.2)	15(55.6)		24(46.2)	48(49.0)	
**Disease, n (%)**				0.736			0.881
AML	127(83.6)	10(76.9)	22(81.5)		44(84.6)	82(83.7)	
MDS	25(16.4)	3(23.1)	5(18.5)		8(15.4)	16(16.3)	
**Remission status at time of HCT** **n (%)**				0.201			0.269
CR1	103(67.8)	6(46.1)	18(66.7)		31(59.6)	71(72.5)	
≥CR2	27(17.8)	4(30.8)	2(7.4)		11(21.2)	15(15.3)	
No CR	22(14.4)	3(23.1)	7(25.9)		10(19.2)	12(12.2)	
**DRI-R, n (%)**				0.564			0.038
Low/Intermediate	94(61.8)	8(61.5)	14(51.9)		26(50.0)	66(67.3)	
High/Very high	58(38.2)	5(38.5)	13(48.1)		26(50.0)	32(32.7)	
**HLA match, n (%)**				0.807			0.210
MMRD/MMUD	127(83.6)	11(84.6)	22(81.5)		41(78.8)	85(86.7)	
MRD/MUD	25(16.4)	2(15.4)	5(18.5)		11(21.2)	13(13.3)	
**Donor sex, n (%)**				0.090			0.145
Male	103(67.8)	11(84.6)	15(55.6)		39(75.0)	62(63.3)	
Female	49(32.2)	2(15.4)	12(44.4)		13(25.0)	36(36.7)	

*Abbreviations* *DTA*, *DNMT3A*, *TET2*, and *ASXL1* mutations; MRD, measurable residual disease; AML, acute myeloid leukemia; MDS, myelodysplastic syndrome; CR, complete remission; CR1, first complete remission; CR2, second complete remission; DRI-R, refined disease risk index; MMRD, mismatched related donor, MMUD, mismatched unrelated donor; MRD, matched related donor; MUD, matched unrelated donor

### Landscape of trackable genes using ddPCR and detection results

There were 54 myeloid neoplasm-associated mutations or fusion genes as trackable molecular targets after allo-HSCT (Supplementary Table 1), with a median number of 2 molecular targets per patient (range 1 to 7). Among the 152 patients, the most frequently detected genes at diagnosis were *WT1* (*n* = 36), *NRAS* (*n* = 25), *FLT3-ITD* (*n* = 24), *NPM1* (*n* = 23), *DNMT3A* (*n* = 20), *CEBPA* (*n* = 18), *TET2*, *RUNX1-RUNX1T* or *IDH2* (*n* = 17), *TP53* (*n* = 13), *RUNX1* (*n* = 10), *IDH1*, *MLL-PTD* or *c-KIT* (*n* = 9), *BCOR* or *U2AF1* (*n* = 8), *CBFβ-MYH11* or *GATA2* (*n* = 7), *KRAS* or *ASXL1* (*n* = 6), *PTPN11*, *FLT3-TKD*, *EZH2*, *CSF3R*, *MLL-ELL* or *STAG2* (*n* = 5).

Fifty-eight patients (38.2%) were MRD positive within 4 months after allo-HSCT, with a median VAF of 0.198%. The most commonly detected posttransplant MRD + targets were *RUNX1-RUNX1T1* (*n* = 11), *TET2* (*n* = 10), *TP53* (*n* = 6), *WT1* (*n* = 5), *RUNX1* or *CBFβ-MYH11* (*n* = 4), and *NRAS*, *NPM1* or *U2AF1* (*n* = 3). Additionally, among targets with a frequency ≥ 5, genes with a relatively high posttransplant MRD + detection ratio (posttransplant /diagnosis) included *RUNX1-RUNX1T1* (detection ratio: 65%), *TET2* (58%), *CBFβ-MYH11* (57%), *TP53* (46%), *RUNX1* (40%), *U2AF1* (38%), and *ASXL1* (33%).

### Prognostic effect of ddPCR-MRD

The median follow-up time of our cohort was 23.1 months (range 5.9–49.6 months) after allo-HSCT. Through analysis of a competing risk model, the overall CIR for patients who were MRD positive after allo-HSCT was higher than for MRD negative patients (33.5% vs. 10.3%, *P* < 0.001), while the NRM of these two groups was similar (6.7% vs. 4.4%, *P* = 0.541). With respect to RFS and OS, we found that MRD-positive patients had significantly inferior OS and RFS compared to MRD-negative patients (RFS: 59.8% vs. 83.8%, *P* < 0.001; OS: 64.3% vs.91.0%, *P* < 0.001). The comparison of CIR, RFS, and OS in the two groups of patients is shown in Supplementary Fig. 1.

### Prognostic effect of *DTA* and non-*DTA*genes as MRD markers

**Fig. 2:**
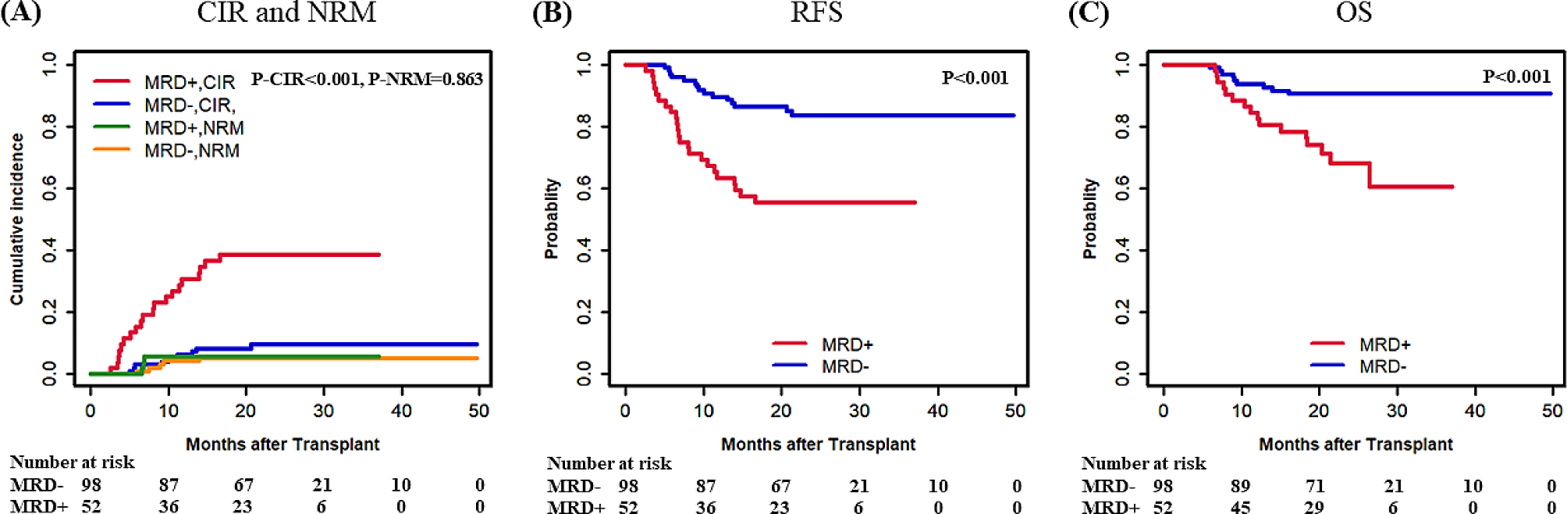
CIR, NRM, RFS, and OS for patients who were MRD positive compared with MRD negative in non-*DTA* genes by ddPCR after allo-HSCT (150 patients). (**A**) CIR and NRM by competing risk analysis for MRD-positive (*n* = 52) and MRD-negative (*n* = 98) patients. (**B**, **C**) RFS and OS by Kaplan-Meier method for MRD-positive (*n* = 52) and MRD-negative (*n* = 98) patients

We further analysed the prognostic impact of *DTA* and non-*DTA* genes to determine whether *DTA* mutations are suitable as MRD markers for predicting outcome after allo-HSCT. A total of 40 patients had at least one *DTA* mutation, while 150 patients had at least one non-*DTA* gene. The *DTA* and non-*DTA* genes were then grouped separately. Among patients with *DTA* genes, 13 patients (32.5%) were detected as MRD + after HSCT, including 10 *TET2* mutations, 2 *DNMT3A* mutations, and 2 *ASXL1* mutations, while in the non-*DTA* gene group, 52 patients (34.7%) were detected as MRD + after allo-HSCT. The CIR (23.1% vs. 14.8%, *P* = 0.571), RFS (69.2% vs. 81.5%, *P* = 0.453), and OS (76.9% vs. 85.2%, *P* = 0.537) (Supplementary Fig. 2) after allo-HSCT were not significantly different between the *DTA* MRD + group and the *DTA* MRD- group, suggesting that *DTA* genes may not have a predictive effect and are not suitable as monitoring markers after transplantation. However, in the non-*DTA* group, patients with MRD + had a higher CIR (38.7% vs. 9.7%, *P* < 0.001) and inferior RFS (55.5% vs. 83.7%, *P* < 0.001) and OS (60.5% vs. 90.5%, *P* < 0.001) (Fig. 2) compared to MRD- patients.

**Fig. 3:**
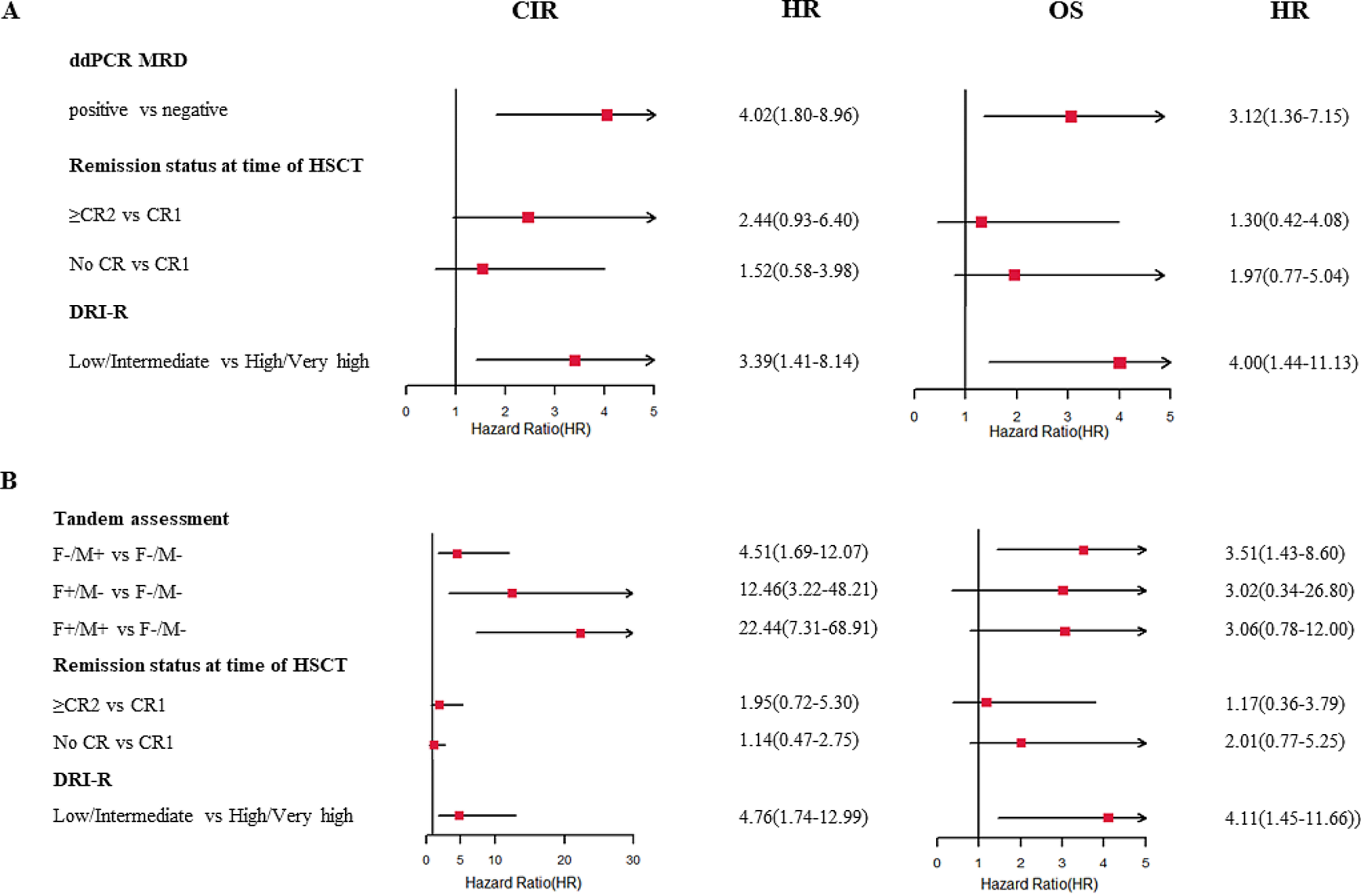
Forest plot showing the first (**A**) and second (**B**) multivariate analysis for CIR and OS

The univariate analysis revealed that ddPCR-MRD + of non-*DTA* genes and high/very high DRI-R were linked to increased CIR, inferior RFS and inferior OS. The remission status of ≥ CR2 at allo-HSCT was associated with increased CIR and inferior RFS, and no CR was associated with an increased risk of CIR and NRM, along with inferior RFS and OS (Table 2). In patients with *DTA* genes, the univariate analysis revealed no significant risk factors associated with these clinical outcomes (Supplementary Table 2). The multivariate analysis model (Fig. 3; Table 3) showed that ddPCR-MRD + in non-*DTA* genes was an independent prognostic factor for CIR (HR, 4.02; 95% CI, 1.80 to 8.96; *P* < 0.001), RFS (HR, 2.92; 95% CI, 1.51 to 5.68; *P* = 0.002) and OS (HR, 3.12; 95% CI, 1.36 to 7.15; *P* = 0.007). Additionally, a higher risk level of DRI-R significantly correlated with higher CIR (HR, 3.39; 95% CI, 1.41 to 8.14; *P* = 0.006), worse RFS (HR, 4.16; 95% CI, 1.86 to 9.34; *P* = 0.001) and worse OS (HR, 4.00; 95% CI, 1.44 to 11.13; *P* = 0.008). The PPV and NPV of the ddPCR-MRD status for predicting relapse in patients with non-*DTA* genes were 38.5% and 90.8%, respectively, with an overall accuracy of 64.6%. The lower PPV compared to NPV is generally considered to be the fact that patients who tested positive for MRD after transplantation received preemptive treatment to eliminate remaining disease, which can prevent relapse in some patients.

**Table 2:** Univariate analysis for CIR, NRM, RFS, and OS in patients with non-*DTA* genes

Variables	n	CIR		NRM		RFS		OS	
		HR (95% CI)	*P* Value	HR (95% CI)	*P* Value	HR (95% CI)	*P* Value	HR (95% CI)	*P* Value
**ddPCR MRD**									
Negative	98	1.00[Reference]		1.00[Reference]		1.00[Reference]		1.00[Reference]	
Positive	52	3.97(1.81–8.71)	< 0.001	1.14(0.27–4.75)	0.860	3.58(1.87–6.87)	< 0.001	3.80(1.67–8.61)	0.001
**Tandem assessment**							< 0.001		0.011
F-/M-	93	1.00[Reference]		1.00[Reference]		1.00[Reference]		1.00[Reference]	
F-/M+	43	5.73(2.23–14.70)	< 0.001	1.49(0.36–6.25)	0.583	3.60(1.72–7.54)	0.001	3.88(1.61–9.38)	0.003
F+/M-	6	9.83(2.54-38.00)	< 0.001	N		4.83(1.36–17.13)	0.015	1.74(0.22–13.98)	0.600
F+/M+	8	25.30(7.24–88.50)	< 0.001	N		12.80(4.77–34.36)	< 0.001	5.54(1.47–20.92)	0.012
**Remission status at time of HSCT**							< 0.001		0.002
CR1	102	1.00[Reference]		1.00[Reference]		1.00[Reference]		1.00[Reference]	
≥CR2	26	2.66(1.12–6.28)	0.026	1.93(0.18–20.70)	0.590	2.42(1.07–5.48)	0.034	1.29(0.42-4.00)	0.661
No CR	22	3.36(1.38–8.16)	0.008	12.37(2.36–64.90)	0.003	5.27(2.53–10.99)	< 0.001	4.50(1.89–10.73)	0.001
**HLA match, n (%)**									
MMRD/MMUD	126	1.00[Reference]		1.00[Reference]		1.00[Reference]		1.00[Reference]	
MRD/MUD	24	0.85(0.30–2.43)	0.760	N		0.60(0.21–1.69)	0.332	0.66(0.20–2.22)	0.506
**DRI-R**									
Low/Intermediate	92	1.00[Reference]		1.00[Reference]		1.00[Reference]		1.00[Reference]	
High/Very high	58	3.97(1.81–8.71)	< 0.001	11.60(1.47–92.10)	0.020	5.51(2.67–11.35)	< 0.001	5.76(2.30-14.43)	< 0.001

*Abbreviations* ddPCR, droplet digital PCR; MRD, measurable residual disease; CIR, cumulative incidence of relapse; NRM, non-relapse mortality; RFS, relapse-free survival; OS, overall survival; HR, hazard ratio; CI, confidence interval; F+/M+, MFC MRD-positive and non-*DTA* MRD-positive; F-/M+, MFC MRD-negative and non-*DTA* MRD-positive; F+/M-, MFC MRD-positive and non-*DTA* MRD-negative; F-/M-, MFC MRD-negative and non-*DTA* MRD-negative; CR, complete remission; CR1, first complete remission; CR2, second complete remission; MMRD, mismatched related donor, MMUD, mismatched unrelated donor; MRD, matched related donor; MUD, matched unrelated donor; DRI-R, refined disease risk index

**Table 3:** Multivariate analysis for CIR, NRM, RFS, and OS in patients with non-*DTA* genes

Variables	n	CIR		NRM		RFS		OS	
		HR (95% CI)	*P* Value	HR (95% CI)	*P* Value	HR (95% CI)	*P* Value	HR (95% CI)	*P* Value
**ddPCR MRD**									
Negative	98	1.00[Reference]				1.00[Reference]		1.00[Reference]	
Positive	52	4.02(1.80–8.96)	< 0.001			2.92(1.51–5.68)	0.002	3.12(1.36–7.15)	0.007
**Remission status at time of HSCT**							0.052		0.366
CR1	102	1.00[Reference]		1.00[Reference]		1.00[Reference]		1.00[Reference]	
≥CR2	26	2.44(0.93–6.40)	0.071	2.41(0.27–21.30)	0.430	2.28(1.00-5.23)	0.051	1.30(0.42–4.08)	0.652
No CR	22	1.52(0.58–3.98)	0.390	5.16(0.67-40.00)	0.120	2.27(1.03–5.01)	0.043	1.97(0.77–5.04)	0.157
**DRI-R**									
Low/Intermediate	92	1.00[Reference]		1.00[Reference]		1.00[Reference]		1.00[Reference]	
High/Very high	58	3.39(1.41–8.14)	0.006	5.78(0.68–49.30)	0.110	4.16(1.86–9.34)	0.001	4.00(1.44–11.13)	0.008

*Abbreviations* ddPCR, droplet digital PCR; MRD, measurable residual disease; CIR, cumulative incidence of relapse; NRM, non-relapse mortality; RFS, relapse-free survival; OS, overall survival; HR, hazard ratio; CI, confidence interval; CR, complete remission; CR1, first complete remission; CR2, second complete remission; DRI-R, refined disease risk index

We further established internal validation at the First Affiliated Hospital of Zhejiang University School of Medicine and external validation at Other Hospitals to better prove feasibility of using ddPCR to detect non-*DTA* genes for predicting prognosis after transplantation. The results of internal validation showed that MRD positivity were significantly associated with higher CIR, lower RFS and OS (*P* < 0.001) (Supplementary Fig. 3). In the external validation cohort, it was observed that MRD-positive patients showed a higher CIR compared to MRD-negative patients (*P* = 0.049), and their RFS demonstrated a borderline significant difference compared to MRD-negative patients (*P* = 0.067). The absence of obviously differences (*P* = 0.325) in OS between the two groups may be attributed to the relatively small sample size in this subset (Supplementary Fig. 4). Overall, the findings from both validation groups strongly indicate a significant association between MRD-positive and higher CIR, in line with previous observations.

### Tandem assessment of the prognostic effect of non-*DTA* genes using ddPCR and MFC

**Fig. 4:**
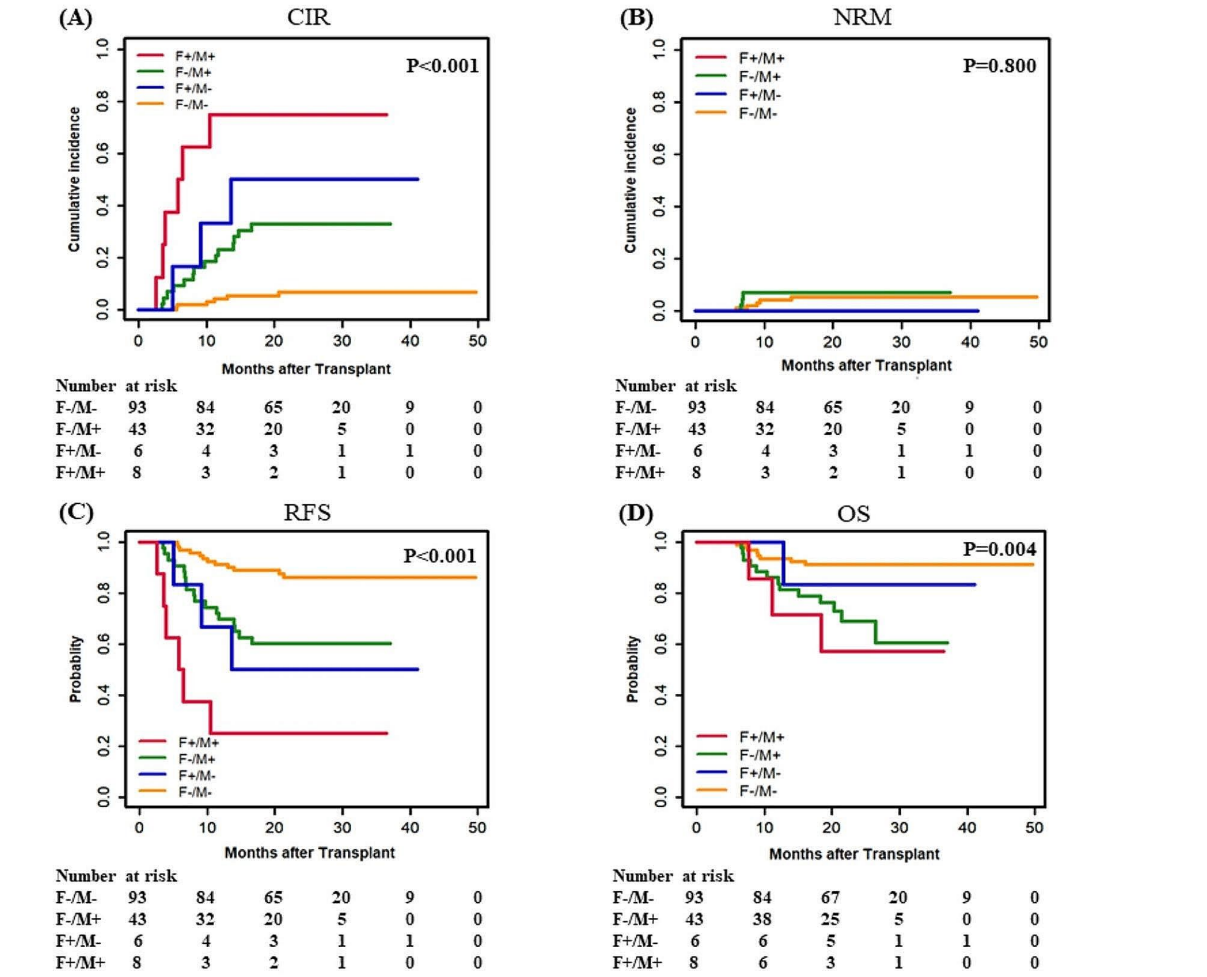
CIR, RFS, and OS for patients who were F+/M+, F-/M+, F+/M-, and F-/M- in non-*DTA* genes after allo-HSCT by tandem assessment (150 patients). (**A**, **B**) CIR and NRM by competing risk analysis for F+/M+ (*n* = 8), F-/M+ (*n* = 43), F+/M- (*n* = 6), and F-/M- (*n* = 93) patients. (**C**, **D**) RFS and OS by Kaplan-Meier method for F+/M+ (*n* = 8), F-/M+ (*n* = 43), F+/M- (*n* = 6), and F-/M- (*n* = 93) patients

To more accurately identify patients who are at high risk of relapse, we performed a tandem assessment of MRD detection using both MFC and ddPCR in patients with non-*DTA* genes. We divided these patients into four groups: ①MFC MRD-positive and non-*DTA* MRD-positive (F+/M+, *n* = 8), ②MFC MRD-negative and non-*DTA* MRD-positive (F-/M+, *n* = 43), ③MFC MRD-positive and non-*DTA* MRD-negative (F+/M-, *n* = 6), and ④MFC MRD-negative and non-*DTA* MRD-negative (F-/M-, *n* = 93). Our analysis showed that patients with F+/M + had the highest CIR (F+/M + 75.0% vs. F+/M- 50.0% vs. F-/M + 32.9% vs. F-/M- 6.9%, *P* < 0.001), the worst RFS (F+/M + 25.0% vs. F+/M- 50.0% vs. F-/M + 60.1% vs. F-/M- 86.1%, *P* < 0.001) and the worst OS (F+/M + 57.1% vs. F+/M- 83.3% vs. F-/M + 60.4% vs. F-/M- 91.1%, *P* = 0.004) compared to the other groups, while patients with F-/M- had the most favorable outcomes in terms of relapse, RFS and OS (Fig. 4). NRM did not differ between the four groups. We conducted a multivariate analysis again on the tandem assessment, DRI-R and remission status at the time of HSCT, and the results showed that the F+/M + group (HR, 22.44; 95% CI, 7.31 to 68.91; *P* < 0.001), the F+/M- group (HR, 12.46; 95% CI, 3.22 to 48.21; *P* < 0.001), and the F-/M + group (HR, 4.51; 95% CI, 1.69 to 12.07; *P* = 0.003) had a significantly increased risk of relapse after allo-HSCT compared to the F-/M- groups (Fig. 3; Table 4). The DRI-R level remained statistically significant after this multivariate analysis: a higher risk level of DRI-R was associated with increased CIR and inferior RFS and OS. The PPV and NPV of MFC alone for predicting relapse were 64.3% and 85.3%, with an overall accuracy of 74.5%. While the PPV and NPV for combined detection exhibited higher PPV and NPV at 75.0% and 93.5%, resulting in an improved overall accuracy of 84.3%. Notably, the false negative rate in the F-/M + group was 10.3%, which was lower than the 14.7% observed in the F- group. This combined approach demonstrated superior diagnostic performance compared to either of the techniques used in isolation.

**Table 4:** Multivariate analysis for CIR, RFS, and OS in patients with non-*DTA* genes after introduction of MFC for tandem assessment

Variables	n	CIR		RFS		OS	
		HR (95% CI)	*P* Value	HR (95% CI)	*P* Value	HR (95% CI)	*P* Value
**Tandem assessment**					< 0.001		0.048
F-/M-	93	1.00[Reference]		1.00[Reference]		1.00[Reference]	
F-/M+	43	4.51(1.69–12.07)	0.003	2.85(1.39–6.30)	0.005	3.51(1.43–8.60)	0.006
F+/M-	6	12.46(3.22–48.21)	< 0.001	7.19(1.84–28.02)	0.004	3.02(0.34–26.80)	0.321
F+/M+	8	22.44(7.31–68.91)	< 0.001	9.97(3.52–28.22)	< 0.001	3.06(0.78-12.00)	0.108
**Remission status at time of HSCT**					0.169		0.358
CR1	102	1.00[Reference]		1.00[Reference]		1.00[Reference]	
≥CR2	26	1.95(0.72–5.30)	0.190	1.94(0.82–4.59)	0.132	1.17(0.36–3.79)	0.795
No CR	22	1.14(0.47–2.75)	0.780	1.86(0.82–4.21)	0.135	2.01(0.77–5.25)	0.152
**DRI-R**							
Low/Intermediate	92	1.00[Reference]		1.00[Reference]		1.00[Reference]	
High/Very high	58	4.76(1.74–12.99)	0.002	5.32(2.26–12.55)	< 0.001	4.11(1.45–11.66)	0.008

*Abbreviations* CIR, cumulative incidence of relapse; RFS, relapse-free survival; OS, overall survival; HR, hazard ratio; CI, confidence interval; F+/M+, MFC MRD-positive and non-*DTA* MRD-positive; F-/M+, MFC MRD-negative and non-*DTA* MRD-positive; F+/M-, MFC MRD-positive and non-*DTA* MRD-negative; F-/M-, MFC MRD-negative and non-*DTA* MRD-negative; CR, complete remission; CR1, first complete remission; CR2, second complete remission; DRI-R, refined disease risk index

## Discussion

This retrospective cohort study was designed to assess the prognostic value of MRD detection after allo-HSCT in AML/MDS based on ddPCR. Our study provides evidence that ddPCR applied to MRD detection for non-*DTA* genes after allo-HSCT can effectively identify patients who are at high risk of relapse. In addition, to our knowledge, this study is the first comprehensive investigation to evaluate MRD by combining MFC with ddPCR, and this combination is proven to further improve the accuracy of predicting relapse after HSCT. Patients with negativity of both MFC and ddPCR were identified to have the most favorable outcomes after allo-HSCT.

The selection of ddPCR for MRD monitoring is often driven by its ability to perform absolute quantification of nucleic acids, resulting in increased sensitivity and accuracy of detection with a lower limit of detection of 0.001% compared to MFC and NGS and without the requirement of a standard curve compared to qPCR [37, 38]. Although NGS has the advantage of detecting complete mutations or fusion genes and monitoring clone evolution or the generation of new genes before and after transplantation, it is still challenging due to its time-consuming and overpriced drawbacks, while most relapsed patients have at least one molecular target in common with their initial diagnosis [26, 27, 39], indicating that a large number of allo-HSCT recipients have sufficient targets to trace MRD status by ddPCR after transplantation. Furthermore, ddPCR is more cost-effective and time-efficient than NGS. These characteristics make ddPCR a viable and comparable alternative to NGS in terms of MRD monitoring after transplantation.

The prognostic impact of *DTA* mutations as posttransplant MRD markers is still uncertain. Our study found that 32.5% (*n* = 13) of patients with *DTA* mutations had persistent *DTA* MRD + in posttransplant, especially *TET2* mutation, and that persistent *DTA* mutations had little effect on prognosis. In contrast, after excluding *DTA* mutations, we observed a stronger association between non-*DTA* MRD + and negative prognosis after transplantation. At the end of follow-up, 3 of the 13 patients with *DTA* MRD + relapsed, while 4 of the remaining 27 patients with *DTA* MRD- relapsed. Among the *DTA* MRD - patients who relapsed, three were at a higher risk of DRI-R prior to HSCT and were found to be non-*DTA* MRD + before relapse. Of note, some patients with *DTA* MRD + had relatively high VAF values (> 5%) in CR status, in line with prior reports [22, 23, 40]. Persistent premalignant clone may explain why high VAF values endure after hematopoietic reconstitution without worsening the clinical outcomes of recipients [22–25]. Previous studies have reported that the persistent cells with *DTA* mutations typically have a selective clonal advantage in proliferation over normal stem cells [41–43]. As premalignant clone rather than residual leukemia is hard to completely eliminate during early period after allo-HSCT in some patients, according to our statistical results about *DTA* mutations, we believe that *DTA* genes were not suitable molecular targets for early posttransplant MRD monitoring. However, this result is inconsistent with a study on monitoring MRD by NGS that considered *DTA* mutations to be reliable MRD markers for relapse after transplantation [27]. More studies on the prognostic value of *DTA* mutations in posttransplant period may be necessary to resolve this controversy.

We also found that 62.5% (15/24) of patients with CBF AML could still be detected with CBF MRD + after transplantation, with a median VAF of 0.011% (RUNX1-RUNX1T1: 0.010%, CBFβ-MYH11: 0.026%). Nevertheless, the incidence of relapse in these patients with CBF MRD + after allo-HSCT was low (RUNX1-RUNX1T1: 18.2%, 2/11; CBFβ-MYH11: 25%, 1/4). Meanwhile, we dynamically monitored the CBF levels of these patients and found that a majority of patients persistently had low VAF values of CBF at multiple time points (30–120 days) after transplantation, with a downward trend in VAF values. This seems to imply that extremely low VAF levels of CBF after early posttransplant period are not associated with relapse [44] or that there is an appropriate cut-off value for CBF MRD to predict prognosis. Considering that our sample size was insufficient to assess the prognostic role of posttransplant CBF alone, we did not remove CBF from our MRD targets.

Although MFC as the posttransplant MRD monitoring assay is less sensitive than ddPCR, some studies have revealed that MFC can indeed identify patients at increased risk of relapse after transplantation [11, 12, 45, 46]. Therefore, we combined MFC with ddPCR for tandem MRD assessment to offset the lower PPV of ddPCR and promote the accuracy of predicting relapse. The combined assessment showed that we can more accurately identify patients at high risk of relapse after transplantation when ddPCR and MFC as MRD monitoring methods complement each other. Moreover, the group of patients with F-/M + status commonly received active prophylaxis or preemptive treatment, such as rapid withdrawal of immunosuppressive therapy, donor lymphocyte infusion, hypomethylating agents and targeted drugs (venetoclax, tyrosine kinase inhibitors, FLT3 inhibitors, etc. ), to decrease the risk of relapse [5, 47], which may be the reason for the low PPV of ddPCR in our cohort. Nevertheless, a low PPV also means that the number of relapses is lower than expected, which may have little impact on overall survival of patients. For the clinical treatment and outcomes of patients, a testing tool with a higher NPV will offer greater benefits to transplant recipients.

In conclusion, our study provides a viable option for MRD monitoring in most patients with AML/MDS following allo-HSCT . MRD positivity of non-*DTA* genes detected by ddPCR after transplantation was associated with an increased risk of relapse. Besides, our study is pioneering in the field of posttransplant MRD monitoring, as it represents the first concerted effort to explore the combined use of ddPCR and MFC for prognostic prediction. When ddPCR is combined simultaneously with MFC to monitor posttransplant MRD, it is more accurate in identifying patients at high risk of relapse.

### Electronic supplementary material

Below is the link to the electronic supplementary material.

**Supplementary Material 1: Supplementary Fig. 1**. CIR, NRM, RFS, and OS for patients who were MRD positive compared with MRD negative by ddPCR after allo-HSCT (152 patients). (**A**) CIR and NRM by competing risk analysis for MRD-positive (*n* = 60) and MRD-negative (*n* = 92) patients. (**B**, **C**) RFS and OS by Kaplan-Meier method for MRD-positive (*n* = 60) and MRD-negative (*n* = 92) patients

**Supplementary Material 2: Supplementary Fig. 2**. CIR, NRM, RFS, and OS for patients who were MRD positive compared with MRD negative in *DTA* genes by ddPCR after allo-HSCT (40 patients). (**A**, **B**) CIR and NRM by competing risk analysis for MRD-positive (*n* = 13) and MRD-negative (*n* = 27) patients. (**C**, **D**) RFS and OS by Kaplan-Meier method for MRD-positive (*n* = 13) and MRD-negative (*n* = 27) patients

**Supplementary Material 3: Supplementary Fig. 3**. Internal verification cohort for ddPCR-MRD at the First Affiliated Hospital of Zhejiang University School of Medicine.
**Supplementary Material 4:** **Supplementary Fig. 4**. External verification cohort for ddPCR-MRD at Other Hospitals.

## Data Availability

The datasets generated and analysed during the current study are available from the corresponding author on reasonable request.

## References

[1] Horowitz M, Schreiber H, Elder A, Heidenreich O, Vormoor J, Toffalori C (2018). Epidemiology and biology of relapse after stem cell transplantation. Bone Marrow Transplant.

[2] Ivey A, Hills RK, Simpson MA, Jovanovic JV, Gilkes A, Grech A (2016). Assessment of minimal residual disease in Standard-Risk AML. N Engl J Med.

[3] Platzbecker U, Middeke JM, Sockel K, Herbst R, Wolf D, Baldus CD (2018). Measurable residual disease-guided treatment with azacitidine to prevent haematological relapse in patients with myelodysplastic syndrome and acute myeloid leukaemia (RELAZA2): an open-label, multicentre, phase 2 trial. Lancet Oncol.

[4] Grimwade D, Freeman SD (2014). Defining minimal residual disease in acute myeloid leukemia: which platforms are ready for prime time?. Blood.

[5] Mo XD, Lv M, Huang XJ (2017). Preventing relapse after haematopoietic stem cell transplantation for acute leukaemia: the role of post-transplantation minimal residual disease (MRD) monitoring and MRD-directed intervention. Br J Haematol.

[6] Kayser S, Walter RB, Stock W, Schlenk RF (2015). Minimal residual disease in acute myeloid leukemia–current status and future perspectives. Curr Hematol Malig Rep.

[7] Ladetto M, Böttcher S, Kröger N, Pulsipher MA, Bader P (2019). Methods and role of minimal residual disease after stem cell transplantation. Bone Marrow Transplant.

[8] Walter RB, Gooley TA, Wood BL, Milano F, Fang M, Sorror ML (2011). Impact of pretransplantation minimal residual disease, as detected by multiparametric flow cytometry, on outcome of myeloablative hematopoietic cell transplantation for acute myeloid leukemia. J Clin Oncology: Official J Am Soc Clin Oncol.

[9] Venditti A, Buccisano F, Del Poeta G, Maurillo L, Tamburini A, Cox C (2000). Level of minimal residual disease after consolidation therapy predicts outcome in acute myeloid leukemia. Blood.

[10] Sanchez-Garcia J, Serrano J, Serrano-Lopez J, Gomez-Garcia P, Martinez F, Garcia-Castellano JM (2013). Quantification of minimal residual disease levels by flow cytometry at time of transplant predicts outcome after myeloablative allogeneic transplantation in ALL. Bone Marrow Transplant.

[11] Zhao XS, Liu YR, Zhu HH, Xu LP, Liu DH, Liu KY (2012). Monitoring MRD with flow cytometry: an effective method to predict relapse for ALL patients after allogeneic hematopoietic stem cell transplantation. Ann Hematol.

[12] Díez-Campelo M, Pérez-Simón JA, Pérez J, Alcoceba M, Richtmon J, Vidriales B (2009). Minimal residual disease monitoring after allogeneic transplantation may help to individualize post-transplant therapeutic strategies in acute myeloid malignancies. Am J Hematol.

[13] Coccaro N, Tota G, Anelli L, Zagaria A, Specchia G, Albano F. Digital PCR: a Reliable Tool for analyzing and monitoring hematologic malignancies. Int J Mol Sci. 2020;21(9). 10.3390/ijms21093141 PMC7247671 32365599

[14] Hindson CM, Chevillet JR, Briggs HA, Gallichotte EN, Ruf IK, Hindson BJ (2013). Absolute quantification by droplet digital PCR versus analog real-time PCR. Nat Methods.

[15] Drandi D, Kubiczkova-Besse L, Ferrero S, Dani N, Passera R, Mantoan B (2015). Minimal residual disease detection by Droplet Digital PCR in multiple myeloma, Mantle Cell Lymphoma, and Follicular Lymphoma: a comparison with real-time PCR. J Mol Diagnostics: JMD.

[16] Coccaro N, Anelli L, Zagaria A, Casieri P, Tota G, Orsini P (2018). Droplet Digital PCR is a Robust Tool for monitoring minimal residual disease in adult Philadelphia-positive Acute Lymphoblastic Leukemia. J Mol Diagnostics: JMD.

[17] Bill M, Grimm J, Jentzsch M, Kloss L, Goldmann K, Schulz J (2018). Digital droplet PCR-based absolute quantification of pre-transplant NPM1 mutation burden predicts relapse in acute myeloid leukemia patients. Ann Hematol.

[18] Brunetti C, Anelli L, Zagaria A, Minervini A, Minervini CF, Casieri P (2017). Droplet Digital PCR is a Reliable Tool for monitoring minimal residual disease in Acute promyelocytic leukemia. J Mol Diagnostics: JMD.

[19] Brambati C, Galbiati S, Xue E, Toffalori C, Crucitti L, Greco R (2016). Droplet digital polymerase chain reaction for DNMT3A and IDH1/2 mutations to improve early detection of acute myeloid leukemia relapse after allogeneic hematopoietic stem cell transplantation. Haematologica.

[20] Bussaglia E, Pratcorona M, Carricondo M, Sansegundo L, Rubio MA, Monter A (2020). Application of a digital PCR method for WT1 to myeloid neoplasms in CR and deep ELN WT1 molecular response (< 10 copies). Ann Hematol.

[21] Waterhouse M, Pfeifer D, Duque-Afonso J, Follo M, Duyster J, Depner M (2019). Droplet digital PCR for the simultaneous analysis of minimal residual disease and hematopoietic chimerism after allogeneic cell transplantation. Clin Chem Lab Med.

[22] Jongen-Lavrencic M, Grob T, Hanekamp D, Kavelaars FG, Al Hinai A, Zeilemaker A (2018). Molecular minimal residual disease in Acute myeloid leukemia. N Engl J Med.

[23] Hourigan CS, Dillon LW, Gui G, Logan BR, Fei M, Ghannam J (2020). Impact of conditioning intensity of allogeneic transplantation for Acute myeloid leukemia with genomic evidence of residual disease. J Clin Oncology: Official J Am Soc Clin Oncol.

[24] Tanaka T, Morita K, Loghavi S, Wang F, Furudate K, Sasaki Y (2021). Clonal dynamics and clinical implications of postremission clonal hematopoiesis in acute myeloid leukemia. Blood.

[25] Grimm J, Bill M, Jentzsch M, Beinicke S, Häntschel J, Goldmann K (2019). Clinical impact of clonal hematopoiesis in acute myeloid leukemia patients receiving allogeneic transplantation. Bone Marrow Transplant.

[26] Heuser M, Heida B, Büttner K, Wienecke CP, Teich K, Funke C (2021). Posttransplantation MRD monitoring in patients with AML by next-generation sequencing using DTA and non-DTA mutations. Blood Adv.

[27] Kim HJ, Kim Y, Kang D, Kim HS, Lee JM, Kim M (2021). Prognostic value of measurable residual disease monitoring by next-generation sequencing before and after allogeneic hematopoietic cell transplantation in acute myeloid leukemia. Blood cancer J.

[28] Chen X, Liu L, Zhang A, Yi M, Lan Y, Zheng Z (2022). Droplet digital PCR for genetic mutations monitoring predicts relapse risk in pediatric acute myeloid leukemia. Int J Hematol.

[29] Ruan M, Zhang LL, Li YM, Li DY, Yuan ZY, Zheng ZZ et al. [Detection of BCR-ABL Fusion Gene in Chronic myeloid leukemia by Novel Digital PCR]. Zhongguo Shi Yan xue ye xue Za Zhi. 2023;31(6):1647–56. 10.19746/j.cnki.issn.1009-2137.2023.06.008 38071041

[30] Wood BL. Ten-color immunophenotyping of hematopoietic cells. Curr Protocols Cytometry. 2005;Chap. 6:Unit6.21. 10.1002/0471142956.cy0621s33 18770824

[31] Wood B (2006). 9-color and 10-color flow cytometry in the clinical laboratory. Arch Pathol Lab Med.

[32] Theunissen P, Mejstrikova E, Sedek L, van der Sluijs-Gelling AJ, Gaipa G, Bartels M (2017). Standardized flow cytometry for highly sensitive MRD measurements in B-cell acute lymphoblastic leukemia. Blood.

[33] Wood BL (2020). Acute myeloid leukemia minimal residual disease detection: the difference from Normal Approach. Curr Protocols Cytometry.

[34] Short NJ, Jabbour E, Albitar M, de Lima M, Gore L, Jorgensen J (2019). Recommendations for the assessment and management of measurable residual disease in adults with acute lymphoblastic leukemia: a consensus of north American experts. Am J Hematol.

[35] Griner PF, Mayewski RJ, Mushlin AI, Greenland P (1981). Selection and interpretation of diagnostic tests and procedures. Principles and applications. Ann Intern Med.

[36] Armand P, Kim HT, Logan BR, Wang Z, Alyea EP, Kalaycio ME (2014). Validation and refinement of the Disease Risk Index for allogeneic stem cell transplantation. Blood.

[37] Panuzzo C, Jovanovski A, Ali MS, Cilloni D, Pergolizzi B. Revealing the mysteries of Acute myeloid leukemia: from quantitative PCR through Next-Generation sequencing and systemic metabolomic profiling. J Clin Med. 2022;11(3). 10.3390/jcm11030483 PMC8836582 35159934

[38] Azenkot T, Jonas BA. Clinical impact of measurable residual disease in Acute myeloid leukemia. Cancers. 2022;14(15). 10.3390/cancers14153634 PMC9330895 35892893

[39] Kim T, Moon JH, Ahn JS, Kim YK, Lee SS, Ahn SY (2018). Next-generation sequencing-based posttransplant monitoring of acute myeloid leukemia identifies patients at high risk of relapse. Blood.

[40] Patkar N, Kakirde C, Shaikh AF, Salve R, Bhanshe P, Chatterjee G (2021). Clinical impact of panel-based error-corrected next generation sequencing versus flow cytometry to detect measurable residual disease (MRD) in acute myeloid leukemia (AML). Leukemia.

[41] Challen GA, Sun D, Jeong M, Luo M, Jelinek J, Berg JS (2011). Dnmt3a is essential for hematopoietic stem cell differentiation. Nat Genet.

[42] Moran-Crusio K, Reavie L, Shih A, Abdel-Wahab O, Ndiaye-Lobry D, Lobry C (2011). Tet2 loss leads to increased hematopoietic stem cell self-renewal and myeloid transformation. Cancer Cell.

[43] Shlush LI, Zandi S, Mitchell A, Chen WC, Brandwein JM, Gupta V (2014). Identification of pre-leukaemic haematopoietic stem cells in acute leukaemia. Nature.

[44] Yalniz FF, Patel KP, Bashir Q, Marin D, Ahmed S, Alousi AM (2020). Significance of minimal residual disease monitoring by real-time quantitative polymerase chain reaction in core binding factor acute myeloid leukemia for transplantation outcomes. Cancer.

[45] Shah MV, Jorgensen JL, Saliba RM, Wang SA, Alousi AM, Andersson BS (2018). Early post-transplant minimal residual Disease Assessment improves risk stratification in Acute myeloid leukemia. Biology Blood Marrow Transplantation: J Am Soc Blood Marrow Transplantation.

[46] Bastos-Oreiro M, Perez-Corral A, Martínez-Laperche C, Bento L, Pascual C, Kwon M (2014). Prognostic impact of minimal residual disease analysis by flow cytometry in patients with acute myeloid leukemia before and after allogeneic hemopoietic stem cell transplantation. Eur J Haematol.

[47] Xuan L, Liu Q (2021). Maintenance therapy in acute myeloid leukemia after allogeneic hematopoietic stem cell transplantation. J Hematol Oncol.

