# Supplementary material for: Measurable residual disease monitoring by ddPCR in the early posttransplant period complements the traditional MFC method to predict relapse after HSCT in AML/MDS: a multicenter retrospective study
Source: J Transl Med. 2024 Apr 30;22:410. doi: 10.1186/s12967-024-05114-w (PMC11061929; doi:10.1186/s12967-024-05114-w)

**Supplementary Table 1. Gene targets of AML/MDS panel**

| **Pathway** | **Gene** |
| --- | --- |
| Chromatin/cohesin | *ASXL1, DDX41, EZH2, MLL-X fusions, MLL-PTD, NUP98, RAD21, SETBP1, SETD2, STAG2* |
| DNA methylation | *DNMT3A, IDH1, IDH2, TET2* |
| Signal transduction | *BCR-ABL, BRAF, CBL, CSF3R, FLT3, JAK2, JAK3, KIT, KRAS, MPL, NF1, NRAS,* *PTPN, PTPN11* |
| Spliceosome | *BCOR, BCORL1, SF3B1, SRSF2, U2AF1, ZRSR2* |
| Transcription factors | *RUNX1-RUNX1T, CBFβ-MYH11, CEBPA, ETV6, GATA2, IKZF1, NOTCH1, NOTCH2, RUNX1, TLS-ERG* |
| Nucleophosmin | *NPM1* |
| Tumor suppressor | *PHF6, TP53, WT1* |

**Supplementary Table 2. Univariate analysis for CIR, NRM, RFS, and OS in patients with DTA genes.**

| **Variables** | **n** | **CIR** | | **NRM** | | **RFS** | | **OS** | |
| --- | --- | --- | --- | --- | --- | --- | --- | --- | --- |
|  |  | **HR (95% CI)** | ***P* Value** | **HR (95% CI)** | ***P* Value** | **HR (95% CI)** | ***P* Value** | **HR (95% CI)** | ***P* Value** |
| **ddPCR MRD**  Negative  Positive | 13  27 | 1.00[Reference]  1.53(0.36-6.49) | 0.561 | 1.00[Reference]  2.16(0.15-32.00) | 0.570 | 1.00[Reference]  1.65(0.44-6.13) | 0.453 | 1.00[Reference]  1.60(0.36-7.14) | 0.537 |
| **Remission status at time of HSCT**  CR1  ≥CR2  No CR | 24  6  10 | 1.00[Reference]  1.43(0.14-14.32)  2.48(0.54-11.40) | 0.763  0.239 | 1.00[Reference]  N  N |  | 1.00[Reference]  1.44(0.15-13.80)  4.58(1.09-19.24) | 0.099  0.754  0.038 | 1.00[Reference]  2.13(0.19-23.51)  5.43(0.99-29.72) | 0.141  0.537  0.051 |
| **HLA match, n (%)**  MMRD/MMUD  MRD/MUD | 33  7 | 1.00[Reference]  0.84(0.09-7.52) | 0.870 | 1.00[Reference]  N |  | 1.00[Reference]  0.61(0.08-4.87) | 0.640 | 1.00[Reference]  N |  |
| **DRI-R**  Low/Intermediate  High/Very high | 22  18 | 1.00[Reference]  1.70(0.40-7.34) | 0.478 | 1.00[Reference]  N |  | 1.00[Reference]  2.68(0.67-10.72) | 0.164 | 1.00[Reference]  1.65(0.37-7.36) | 0.514 |

**Abbreviations:** ddPCR, droplet digital PCR; MRD, measurable residual disease; CIR, cumulative incidence of relapse; NRM, non-relapse mortality; RFS, relapse-free survival; OS, overall survival; HR, hazard ratio; CI, confidence interval; CR, complete remission; CR1, first complete remission; CR2, second complete remission; MMRD, mismatched related donor, MMUD, mismatched unrelated donor; MRD, matched related donor; MUD, matched unrelated donor; DRI-R, refined disease risk index.

**Supplementary Figure 1. CIR, NRM, RFS, and OS for patients who were MRD positive compared with MRD negative by ddPCR after allo-HSCT (152 patients).** (A) CIR and NRM by competing risk analysis for MRD-positive (n=60) and MRD-negative (n=92) patients. (B, C) RFS and OS by Kaplan-Meier method for MRD-positive (n=60) and MRD-negative (n=92) patients.


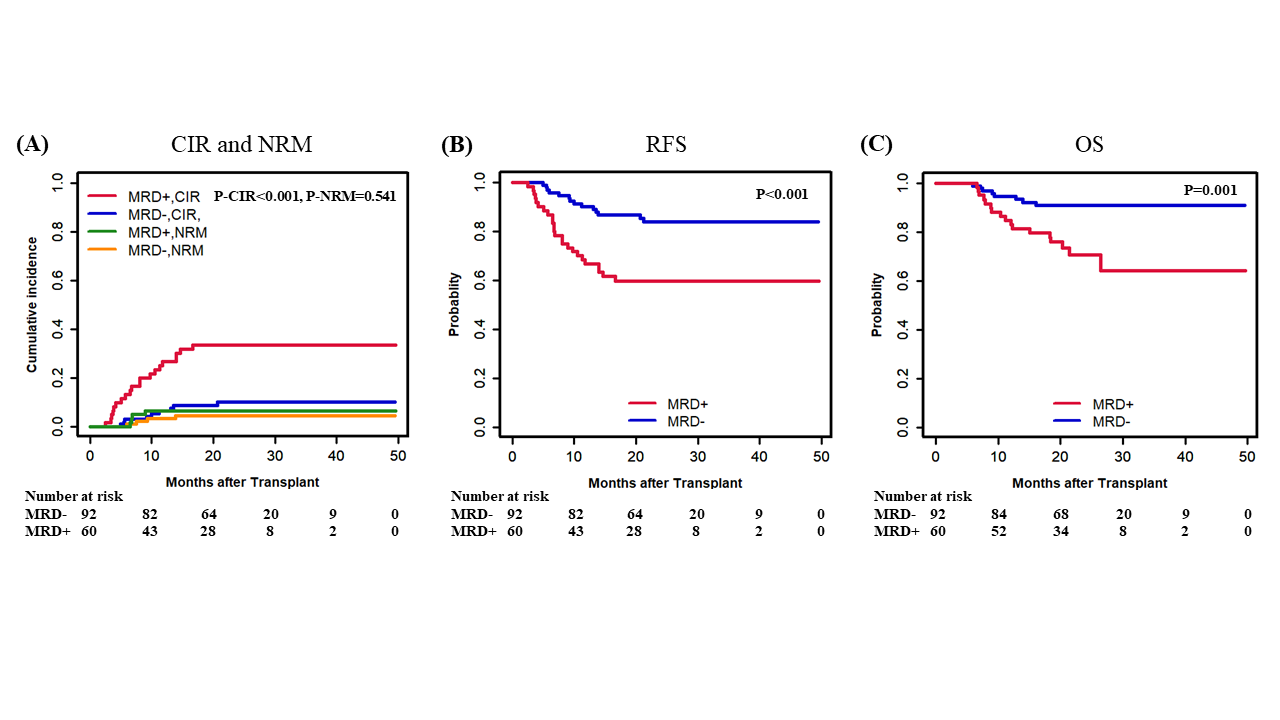


**Supplementary Figure 2. CIR, NRM, RFS, and OS for patients who were MRD positive compared with MRD negative in DTA genes by ddPCR after allo-HSCT (40 patients).** (A, B) CIR and NRM by competing risk analysis for MRD-positive (n=13) and MRD-negative (n=27) patients. (C, D) RFS and OS by Kaplan-Meier method for MRD-positive (n=13) and MRD-negative (n=27) patients.


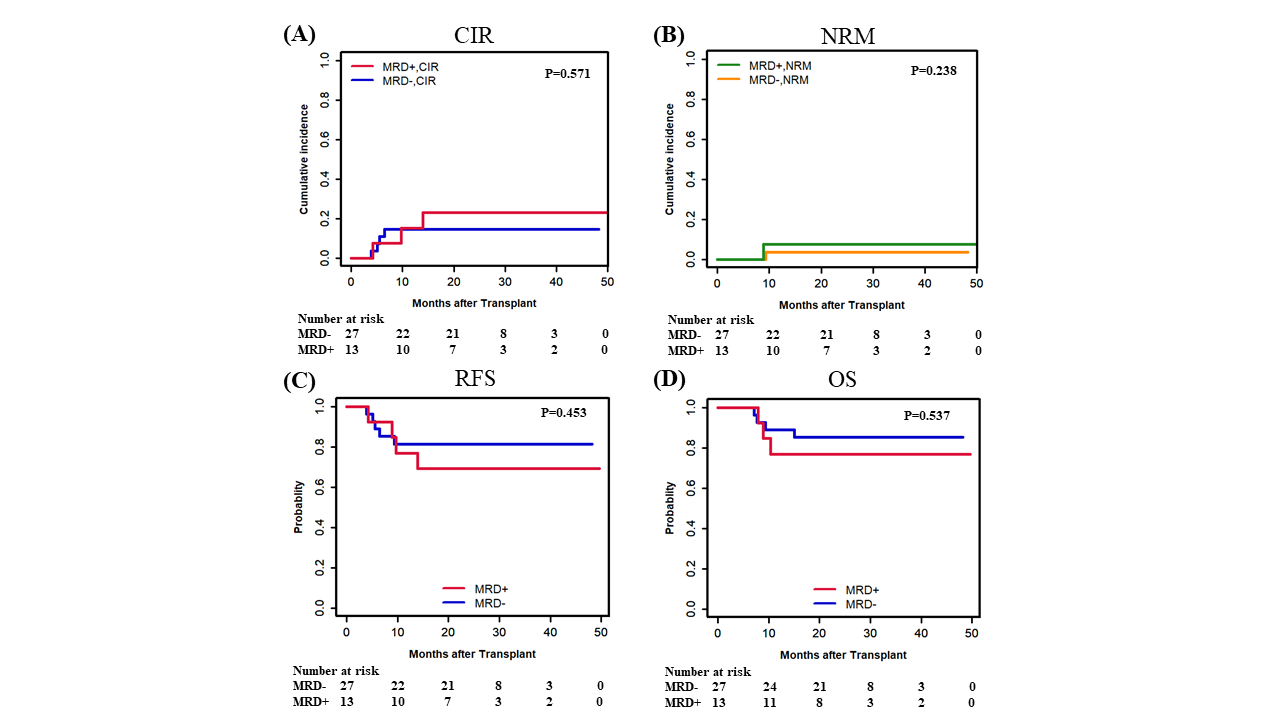


**Supplementary Figure 3. Internal verification cohort for ddPCR-MRD at the First Affiliated Hospital of Zhejiang University School of Medicine**


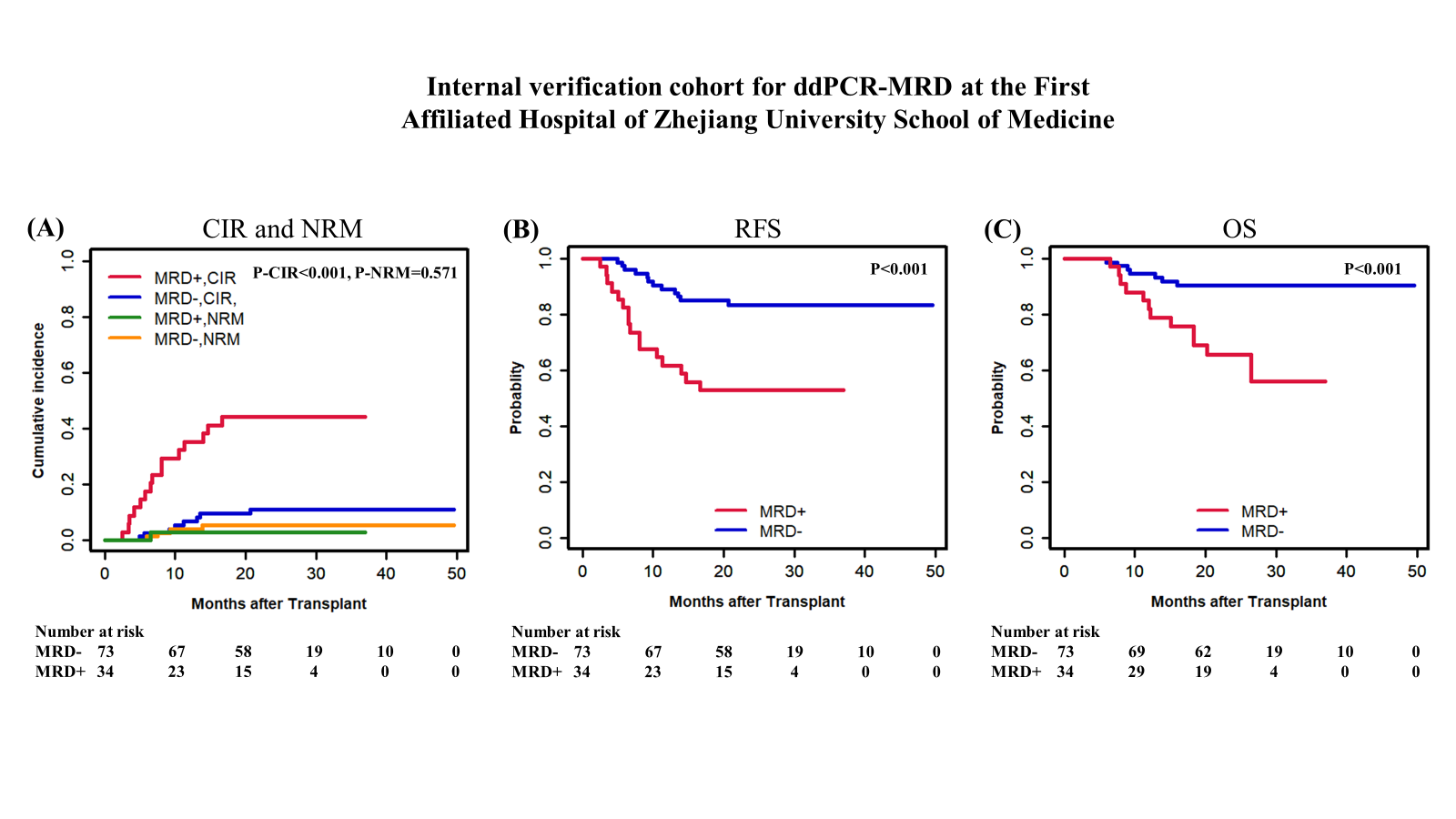


**Supplementary Figure 4. External verification cohort for ddPCR-MRD at Other Hospitals**
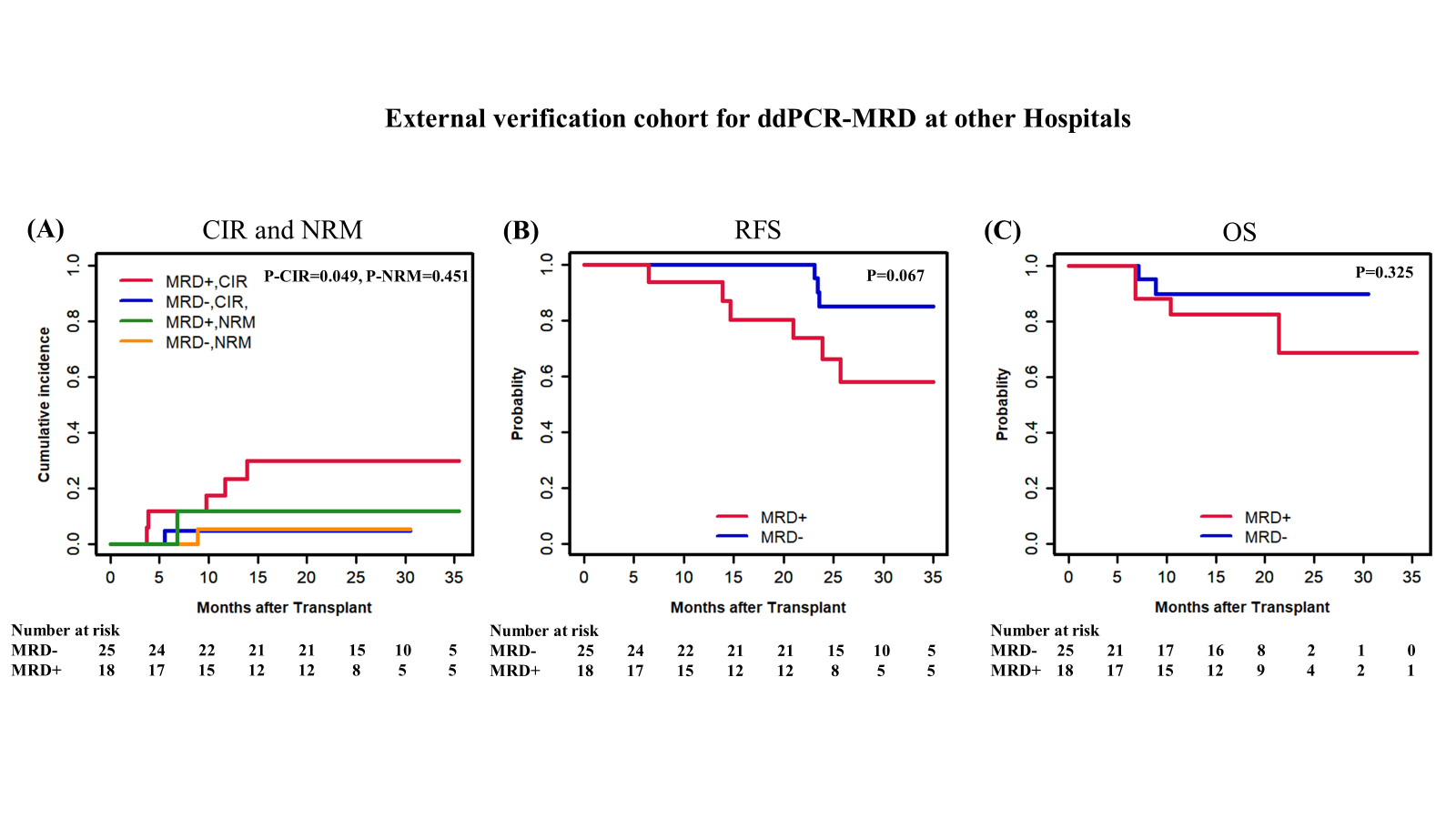

Supplement: Supplementary file 1 — Supplementary Material 1: Supplementary Fig. 1. CIR, NRM, RFS, and OS for patients who were MRD positive compared with MRD negative by ddPCR after allo-HSCT (152 patients). (A) CIR and NRM by competing risk analysis for MRD-positive (n = 60) and MRD-negative (n = 92) patients. (B, C) RFS and OS by Kaplan-Meier method for MRD-positive (n = 60) and MRD-negative (n = 92) patients [file 12967_2024_5114_MOESM1_ESM.docx]
