# Supplementary material for: Measurable residual disease monitoring by ddPCR in the early posttransplant period complements the traditional MFC method to predict relapse after HSCT in AML/MDS: a multicenter retrospective study
Source: J Transl Med. 2024 Apr 30;22:410. doi: 10.1186/s12967-024-05114-w (PMC11061929; doi:10.1186/s12967-024-05114-w)
Supplement: Supplementary file 3 — Supplementary Material 3: Supplementary Fig. 3. Internal verification cohort for ddPCR-MRD at the First Affiliated Hospital of Zhejiang University School of Medicine. Supplementary Material 4: Supplementary Fig. 4. External verification cohort for ddPCR-MRD at Other Hospitals. [file 12967_2024_5114_MOESM3_ESM.docx]

**“****MRD Monitoring by** **Digital Droplet PCR in the Early Posttransplant Period Complements the Traditional** **MFC Method to Predict Relapse after HSCT in AML/MDS: a Multicenter Retrospective Study” Cover Letter**

Dear Editorial Team of [***Journal of Translational Medicine***](https://translational-medicine.biomedcentral.com/),

We would like to submit our manuscript titled " **MRD Monitoring by Digital Droplet PCR in the Early Posttransplant Period Complements the Traditional MFC Method to Predict Relapse after HSCT in AML/MDS: a Multicenter Retrospective Study** " to [***Journal of Translational Medicine***](https://translational-medicine.biomedcentral.com/) as an original article. This work has presented as poster **at the 64th ASH Annual Meeting.** The choice to consider [***Journal of Translational Medicine***](https://translational-medicine.biomedcentral.com/) as the potential platform for disseminating my research stems from its exceptional reputation in the communication between basic and clinical science. The journal is renowned for its commitment to in-depth research and cutting-edge discoveries, making it an ideal venue to present the innovative findings of my study. I am confident that publishing this research in [***Journal of Translational Medicine***](https://translational-medicine.biomedcentral.com/) would provide valuable insights for fellow researchers and contribute to advancing the collective knowledge in this field.

No conflict of interest exists in the submission of this manuscript, and manuscript is approved by all authors for publication. I would like to declare on behalf of my co-authors that the work described was original research that has not been published previously, and not under consideration for publication elsewhere, in whole or in part. All the authors listed have approved the manuscript that is enclosed.

Nowadays, Droplet digital PCR is widely applied to measurable residual disease (MRD) monitoring. However, there are limited studies on the feasibility of ddPCR for MRD monitoring after allogeneic hematopoietic stem cell transplantation (allo-HSCT), especially targeted multiple molecular markers simultaneously. This study aimed to evaluate if post-transplant MRD monitoring by ddPCR can accurately distinguish patients with AML/MDS at high risk of relapse.

Our study has four main advantages:

First, ddPCR is a promising technology for absolute quantification of nucleic acids developed in recent years, with a lower limit of detection (0.001%) compared to multiparameter flow cytometry (MFC) or qPCR. Moreover, it is more time-saving and cost-effective than next-generation sequencing.

Second, we tracked MRD status after allo-HSCT using multiple molecular markers simultaneously rather than specific single genes, which can promote the accuracy of ddPCR to predict relapse and is applicable to most patients with AML/MDS.

Third, considering that the prognostic impact of persistent *DTA* mutations during the posttransplant period remains controversial, our study once again validated the role of DTA mutations and demonstrated that persistent *DTA* mutations after transplantation had little effect on prognosis.

Fourth, this study is the first comprehensive investigation to evaluate MRD by combining MFC with ddPCR, and this combination is proven to further improve the accuracy of predicting relapse after HSCT.

Therefore, our study offered a desirable choice of MRD monitoring for most patients after allo-HSCT, which can provide prognostic information and guide preventive or preemptive intervention to improve overall survival of patients.

We greatly appreciate you for considering our manuscript. Looking forward to your reply. Thanks a lot.

Best wishes,

Yanmin Zhao, Professor/MD/PhD

Bone Marrow Transplantation Center, The First Affiliated Hospital, School of Medicine, Zhejiang University.

No.79 Qingchun Road, Hangzhou, China.

E-mail: zjzhaoyanmin@163.com

Xiaoxia Hu, Professor/MD/PhD

State Key Laboratory of Medical Genomics, Shanghai Institute of Hematology, National Research Center for Translational Medicine, Shanghai Rui Jin Hospital, Shanghai Jiao Tong University School of Medicine.

No.197 Ruijiner Road, Shanghai, China.

E-mail: hu_xiaoxia@126.com

He Huang, Professor/MD/PhD

Bone Marrow Transplantation Center,

The First Affiliated Hospital,

School of Medicine, Zhejiang University.

No.79 Qingchun Road, Hangzhou, China.

E-mail: [huanghe@zju.edu.cn](mailto:huanghe@zju.edu.cn)
