# Supplementary material for: Measurable residual disease monitoring by ddPCR in the early posttransplant period complements the traditional MFC method to predict relapse after HSCT in AML/MDS: a multicenter retrospective study
Source: J Transl Med. 2024 Apr 30;22:410. doi: 10.1186/s12967-024-05114-w (PMC11061929; doi:10.1186/s12967-024-05114-w)
Supplement: Supplementary file 2 — Supplementary Material 2: Supplementary Fig. 2. CIR, NRM, RFS, and OS for patients who were MRD positive compared with MRD negative in DTA genes by ddPCR after allo-HSCT (40 patients). (A, B) CIR and NRM by competing risk analysis for MRD-positive (n = 13) and MRD-negative (n = 27) patients. (C, D) RFS and OS by Kaplan-Meier method for MRD-positive (n = 13) and MRD-negative (n = 27) patients [file 12967_2024_5114_MOESM2_ESM.docx]

Dear Professor Editors and Reviewers:

Enclosed, please find the revised version of our manuscript entitled, “MRD Monitoring by Digital Droplet PCR in the Early Posttransplant Period Complements the Traditional MFC Method to Predict Relapse after HSCT in AML/MDS: a Multicenter Retrospective Study”, which we would like it to be reconsidered for publication in ‘[Journal](https://www.dovepress.com/infection-and-drug-resistance-journal) of Translational Medicine’. We appreciate the opportunity you gave us to revise the manuscript and thank you and the reviewers for the comments. The manuscript has been revised according to reviewers’ and your valuable suggestions. The detailed responses are summarized below.

Looking forward to hearing from you soon.

Sincerely yours,

Corresponding author

Yanmin Zhao, Professor/MD/PhD

Bone Marrow Transplantation Center, The First Affiliated Hospital, School of Medicine, Zhejiang University.

No.79 Qingchun Road, Hangzhou, China.

E-mail: zjzhaoyanmin@163.com

Xiaoxia Hu, Professor/MD/PhD

State Key Laboratory of Medical Genomics, Shanghai Institute of Hematology, National Research Center for Translational Medicine, Shanghai Rui Jin Hospital, Shanghai Jiao Tong University School of Medicine.

No.197 Ruijiner Road, Shanghai, China.

E-mail: hu_xiaoxia@126.com

He Huang, Professor/MD/PhD

Bone Marrow Transplantation Center,

The First Affiliated Hospital,

School of Medicine, Zhejiang University.

No.79 Qingchun Road, Hangzhou, China.

E-mail: [huanghe@zju.edu.cn](mailto:huanghe@zju.edu.cn)

Reviewer #1: In this paper, the authors investigate MRD monitoring in AML and MDS using ddPCR after allo-HSCT. Though it is a standard practice the interesting part of the paper is determining the role of nonDTA genes in MRD monitoring. Further, the authors compare the use of MFC along with ddPCR and investigate the efficiency of the use of dual techniques for MRD detection. The revisions are as follows:

1. The title of the paper needs to be changed for a better representation of the work and so that it reaches a broader reader. MRD full form needs to be written, while the short form of Digital droplet PCR can be used. Further, from the materials method it seems the authors have generated to be used for this paper. If so, then it is not a retrospective study. To my understanding retrospective study is one reanalysis of previously generated data from some other papers. The authors should consider this.

Responses 1: Thanks to the reviewer for the valuable comments. To reach a broader reader for our research, we have revised the title to “Measurable Residual Disease Monitoring by ddPCR in the Early Posttransplant Period Complements the Traditional MFC Method to Predict Relapse after HSCT in AML/MDS: a Multicenter Retrospective Study”. Furthermore, in clinical practice, retrospective research is usually defined as studying the correlation between exposure factors and clinical outcomes in an existing cohort, where the outcome must have already occurred when researchers analyzed the exposure factors and outcomes. In our study, the exposure factor is MRD positive, and outcomes are relapse, OS and RFS. This figure found in WIKIPEDIA shows the description of observational study. Therefore, our study may be more consistent with the retrospective cohort study.

2. The line 182-183 "there was no significant difference in basic characterization……MRD negative patients with DTA genes" not clear.

Responses 2: Thanks to the reviewer for pointing out this error. There may be confusion in the expression. What we mean is that there is no statistical difference in baseline characteristics between the DTA MRD-positive group and DTA MRD-negative group. The data is detailed in Table 1. A modified sentence is presented in revised manuscript.

3. In the 40 patients where MRD was detected with DTA genes, if the same was done with nonDTA genes, how good the prognosis is?

Responses 3: Among the 40 patients in whom MRD was detected with DTA genes, 38 patients had at least one non-DTA gene, including 9 non-DTA MRD-positive and 29 non-DTA MRD-negative. Patients with MRD+ had a higher CIR (66.7 vs. 3.4%, P < 0.001) and inferior RFS (33.3% vs. 89.7%, P < 0.001) and OS (55.6% vs. 89.7%, P = 0.022) compared to MRD- patients. Considering that the sample size was only 38 cases, we select to include these patients into all non-DTA patients for further analysis.

4. Univariate and multivariate analysis needs to be done of DTA genes also to prove the point like that of nonDTA genes.

Responses 4: Thanks for your suggestion, we conducted the univariate analysis for CIR, NRM, RFS, and OS in patients with DTA genes, as shown in Supplementary Table 2. The univariate analysis found no significant risk factors associated with these clinical outcomes, so we did not perform further multivariate analysis for DTA genes.

5. For DTA genes a probable explanation from other mechanistic studies should be given as a reason for DTA gene not able to detect MRD.

Responses 5: We consider premalignant clone that is difficult to completely eliminate by allo-HSCT may explain why DTA genes persists posttransplant and does not affect patient prognosis. This content has presented and highlight with yellow in the Discussion section.

6. Analysis of other databases to link DTA and MRD.

Responses 6: Other papers on DTA and MRD has been analyzed and compared with our study in the Discussion section. We also highlight with yellow in revised manuscript.

7. For use of dual techniques -MFC and ddPCR, the authors should show if the dual technique is used how good it is in detecting false positive as well as false negative data in comparison to either of the techniques alone. In other words, the author should calculate the type I and type II errors. For example, the authors should compare F+ and F- for MRD detection. Then if F+ M+ vs F+ to show how good detection is and F-M+ vs F- to determine false negative. A detailed analysis needs to be done.

Responses 7: The PPV and NPV of ddPCR or MFC alone for predicting relapse were 38.5% and 90.8%, and 64.3% and 85.3%, respectively. While the PPV and NPV for combined detection approach exhibited higher PPV and NPV at 75.0% and 93.5%, resulting in an improved overall accuracy of 84.3%. Notably, the false negative rate in the F-/M+ group was 10.3%, which was lower than the 14.7% observed in the F- group. This content has presented and highlight with yellow in “Tandem assessment of the prognostic effect of non-DTA genes using ddPCR and MFC” of the Result section.

**Reviewer #2:** In the current manuscript, the authors evaluated the feasibility of using droplet digital PCR (ddPCR) to monitor measurable residual disease (MRD) after allogeneic hematopoietic stem cell transplantation (allo-HSCT) in patients with acute myeloid leukemia or high-risk myelodysplastic syndrome. The results showed that ddPCR-MRD, specifically for non-DTA genes, could predict a higher risk of relapse and lower rates of relapse-free survival and overall survival. When combining ddPCR with multiparameter flow cytometry (MFC) , it is more accurate in identifying patients at high risk of relapse. To qualify for publication on the Journal of Translational Medicine, the authors should address the following concerns/suggestions:

1. The authors did not provide the ddPCR-MRD assay methodology information, especially the non-DTA targets and associated primer sequences. This is critical information required for the scientific validity of the current manuscript.

Responses 1: Thanks to the reviewer for this valuable comment. We provide all gene targets for detection of MRD after following your suggestion (Supplementary Table 1). Associated primer sequences were customed based on initial genetic information at diagnosis and were designed and tested by Shanghai Dishuo Beken Biotechnology Co., Ltd. The primers, probe design, and detection were consistent with the company's previously published studies (PMID: 35849248; PMID: 38071041).

2. Do we have enough sample size (152 evaluable subjects) to establish a discovery and validation cohort to better interpret the ddPCR-MRD results? The authors need to revise the statistical analyses or at least discuss the pros and cons of different approaches.

Responses 2: These figures are the ddPCR-MRD validated in two different validation cohorts. The results of internal validation can explain our conclusion well. In the external validation cohort, it was observed that ddPCR MRD-positive patients exhibited a higher CIR compared to MRD-negative patients (P=0.049), and their RFS demonstrated a borderline significant difference compared to MRD-negative patients (P=0.067). The lack of observed differences (P=0.325) in OS between the two groups may be attributed to the relatively small sample size in this subset. Overall, the findings from both validation groups strongly indicate a significant association between ddPCR MRD-positive and higher CIR, in line with previous observations. Further, we have revised the statistical analysis and highlighted with yellow in the manuscript.

3. Elaborate why DTA gene based ddPCR could not detect MRD. Is there any mechanism of action study/publication that supports this conclusion?

Responses 3: We consider premalignant clone that is difficult to completely eliminate by allo-HSCT may explain why DTA genes persists posttransplant and does not affect patient prognosis. Other literature related to DTA and MRD that support this conclusion have been analyzed. This detailed content has presented and highlight with yellow in the Discussion section.
